# A Comparison of Blood-lead Level (BLL) in Opium-dependant Addicts With Healthy Control Group Using the Graphite Furnace/atomic Absorption Spectroscopy (GF-AAS) Followed by Chemometric Analysis

**Published:** 2012-08-30

**Authors:** Mojtaba Amiri, Ramin Amini

**Affiliations:** 1 Young Researchers Club, Islamic Azad University, Shahr-e-Qods Branch, Tehran, IR Iran, P.O. Box 37515-374

**Keywords:** Blood, Lead, Opium, Chemo Metric Analysis

## Abstract

**Background:**

A comparison of oral/inhaled opium addicts with a healthy control group was investigated. Using the graphite furnace atomic absorption spectroscopy (GF-AAS) followed by chemometric analysis, sub-to-low µg L-1 concentrations of blood lead level (BLL) was detected in both the addict and the control groups.

**Materials and Methods:**

In this study, BLL of 78 subjects (Iranian volunteers) in two opium-addicted (patient group) and healthy control groups was evaluated. All the volunteers were men. The patient group was comprised of 39 patients who used opium orally or by inhalation with a mean age of 48.6 ± 7.3 years. The patient group was selected through systematic incidental sampling from 150 orally or by inhalation opium-addicted patients referred to Shariati Hospital located in Tehran .The control group (39 subjects) was matched with the patient group with regard to age and sex and with a mean age of 44.8 ± 5.6 years.

**Results:**

The mean concentration of lead was found to be significantly lower (P = 0.0001) in control group (16.70 ± 12.51 μg/dL) compared to addicts (57.04 ± 46.03 μg/dL). When the addicts were divided into various age groups, there appeared to be a significant difference (p= 0.0451) in blood lead concentration as a function of age, however when the control group was considered, no difference was observed (P = 0.51). Also, a tendency (P = 0.048) towards increasing BLL with respect to BMI was observed due to drug consumption, but there was no significant variation between BLL concentration and BMI when the control group was considered (P = 0.35).

**Conclusions:**

It was observed that the BLL in opium-addicts was significantly higher than that of the healthy control group. The mean difference of both groups was statistically significant

## 1. Introduction

Lead and inorganic lead compounds are found in a variety of commercial and industrial products, including paints, plastics, storage batteries, bearing alloys, insecticides, and ceramics [1][2]. Some of the most important sources of lead in our environment are anthropogenic activities such as burning fossil fuels, mining, contaminated food, industrial emission, soil and various manufacturing processes [3]. Exposure to the above mentioned sources through ingestion, inhalation, or dermal contact can cause significant toxicity [3][4]. Lead poisoning is a medical condition that is caused by increased levels of lead in the blood. Lead may cause irreversible neurological damage as well as renal disease, Coronary artery disease, and reproductive toxicity. Exposure to high level of lead can severely damage the kidney and brain. In addition, nonspecific symptoms similar to lead poisoning such as nonspecific abdominal pain, constipation, irritability, Bone mineral density, muscle aches, headache, and anorexia and so on are found in opium addicted patients [5][6][7][8][9][10][11][12][13]. Normal BLL ranges differ throughout the world [4]. A study in Tehran showed 35.5% of the bus drivers had a BLL of more than 50 µg/dl. Abdollahi et al. showed that 94% of copy center workers had a BLL > 49.9 µg/dl [14]. Another study in paint factory indicated that the mean BLL of employees (50.71µg/dl) was significantly higher than the control group (20.44µg/dl) [15]. However, Lead poisoning of opium addicted patients due to opium impurities is one of the major health problems in Middle East countries such as Iran [4]. Toxic metals in opium and other drugs are very important in forensic and clinical toxicology. The presence of lead in opium considered by Aghaee-Afshar, revealed that salesmen and smugglers may add lead to opium to increase its weight for more profit [16]. In a similar study, a comparison of serum lead level in oral opium addicts with healthy control group was made. However, there was no significant correlation with duration of opium ingestion in the patient group was found [4]. Some cases of lead poisoning in addicted patients with abdominal pain were reported by Algora et al. Masoodi et al. reported three cases of lead poisoning in Iran [17]. Antonini et al. reported lead poisoning during heroin addiction [18]. Another study showed five cases of lead toxicity from self-injection of lead and opium [19]. The International Agency for Research on Cancer (IARC) has determined that inorganic lead is probably carcinogenic to human. Thus, the determination of lead in environmental and biological samples at ultra-trace level especially in blood serum is of great importance [5][7][20].

In this research, a comparison between the ingested/inhaled opium addicts and healthy control group was made. Using the graphite furnace atomic absorption spectroscopy (GF-AAS) followed by chemometric analysis, BLL of 78 subjects in two patient and healthy control group was evaluated. The patient group was selected from 150 of orally/inhaled opium addicts based on their self declaration which had nonspecific abdominal pain referred to Hospital.

## 2. Materials and Methods

The entire reagents used were of analytical reagent grade. Diamonium hydrogen phosphate and Chloroform were purchased from Merck (Darmstadt, Germany). Triton X-100 was purchased from Fluka (Buchus, Switzerland). Quality control material (QCM), Lot No. MR4206, was obtained from Seronorm^TM^ Trace Elements Whole Blood (Norway) with a certified value of 21 ± 4 µgL^-1^ of lead. Plastic tubes containing lithium heparin (Vocuette, Geiner Labortechnik, Kremsmünster, Austria) was used as blood collection vessels. Standard stock solution of lead (II) was prepared by dissolving the appropriate amount of lead nitrate in deionized water containing 1 mL of concentrated HNO3. The working solutions were prepared by the dilution of the stock solution. A 24 µgL^-1^ stock solution of QCM was prepared by dissolving the substance in 5 ml deionized water and Triton X-100 (0.1%). A solution of (NH_4_)H_2_PO_4_ 1% (W/V) dissolved in Triton solution X-100 was used as a modifier. Varian Spectr AA 220 model GTA 110 atomic absorption spectrometer (Mulgrave, Australia) with graphite furnace (GFA-Varian) pyrolytically coated with graphite tubes, a Varian lead hallow cathode lamp as radiation source (Mulgrave, Australia) at the 283.3 nm wavelength with a slit width of 0.5 nm, 5 mA current and deuterium background corrector were used for measurements. The temperature program used to determine the lead by GF-AAS is shown in Table 1. Argon was used as the inert gas at 300 mLmin^-1^ (drying and ashing) except during the atomization step; the flow was stopped and 2000 mLmin^-1^ while cleaning condition. In this study, BLL of 78 subjects (Iranian volunteers) in two opium-addicted (patient group) and healthy control groups was evaluated. All the volunteers were men. The patient group was comprised of 39 patients who used opium orally or by inhalation with a mean age of 48.6 ± 7.3 years. The patient group was selected through systematic incidental sampling from 150 orally or by inhalation opium-addicted patients referred to Shariati Hospital located in Tehran .The control group (39 subjects) was matched with the patient group with regard to age and sex and with a mean age of 44.8 ± 5.6 years. Both the patient and the control groups were among the middle to high socioeconomic people with urban dietary habits. Serum samples were obtained by venipuncture, collected in plastic tubes containing lithium heparin and stored at -20ºC until the analysis. The direct injection method was successfully applied for the determination of selenium in serum samples that were collected from 140 different persons in two patient and control groups. 1 mL of the serum samples of all the 140 subjects from both the patient and healthy control group was collected and were diluted with Triton X-100 (dilution factor was 5). Then, the concentration of selenium was calculated using the calibration curve and the appropriate dilution factor was applied in the calculations.

**Table 1:** Furnace optimized parameters for analysis of lead in blood by GFAAS.

**Step**	**Temperature, ºC**	**Time, s**	**Argon flow-rate, mLmin^-1^**
Drying	85	5.0	3000
Pre last drying	95	40.0	3000
Post last drying	120	10.0	3000
Ashing	600	5.0	3000
Ashing	600	2.1	3000
Gas stop	600	2.1	0
Ramp stop	2200	1.0	0
Atomization	2200	2.0	0
Tube clean	2400	2.0	3000

The normality of the distributions was evaluated by the Chi square test. Group means comparisons were tested for significance by Student’s t-test. A variance analysis (ANOVA) was performed in order to compare drug consumption, age and body mass index (BMI) with the evaluated parameter. All results were expressed as mean values ± SD, statistical significance was defined as P <0.05. For study the changes with age and BMI, the individuals were divided into three age (20-40, 41-60 and 61-80 years) (Table 3) and BMI (12-19, 20-27 and 28-35 kg/m2) (Table 4) groups and statistical evaluation was carried out by using the SPSS 11.5 version for windows.

**Table 2:** Analytical Performance Data of the Method for the Determination of Lead

**Element**	**Linear range, µg L^-1^**	**Coefficient of determination, R^2^**	**LOD, µg L^-1^**	**LOQ, µg L^-1^**	**RSD, % (n=6)**
Pb	5-75	0.9984	1	3.33	2.3

**Table 3:** Blood lead levels (mean ± SD) for three age groups.

**Age groups, y**	**Addicts, μg/dL**	**Controls, μg/dL**	**P value**
20-40	57.01 ± 33.86	19.07 ± 13.73	0.0005
41-60	76.04 ± 55.33	13.30 ± 10.18	0.0003
61-80	30.48 ± 38.76	17.39 ± 13.49	0.0045

**Table 4:** Blood Lead Levels (mean ± SD) for Three BMI Groups

**Age groups, y**	**Addicts, μg/dL**	**Controls, μg/dL**	**P value**
12-19	49.64 ± 39.13	16.41 ± 11.61	0.009
20-27	61.38 ± 43.98	13.32 ± 11.70	0.003
28-35	59.76 ± 56.29	20.66 ± 14.59	0.008

## 3. Results

Different instrumental conditions for the determination of lead were optimized and the optimum values are shown in Table 1. In order to evaluate the linearity of the direct GF-AAS method, a calibration curve over a concentration range of µg L^-1^ of lead was obtained. All the experiments were carried out in triplicate at each concentration point. This gave a linear regression with a coefficient of determination (R^2^) of 0.9984. Linear dynamic range (LDR), coefficient of determination (R^2^), limit of detection (LOD), limit of quantification (LOQ) and relative standard deviation (RSD %) are shown in Table 2. LOD and LOQ were defined as three (C_m_=3δ_b_/m) and ten (C_m_=10δ_b_/m) times of the base-line noise, respectively where C_m_ is limit of detection, δ_b_ is the standard deviation of the blank signal, and m is the slope of the regression line. Six repeated determinations of a standard solution containing 50 µg L^-1^ of Pb were done to calculate the precision (RSD %). The accuracy of the GF-AAS method was checked by recovery studies and the analysis of a blood quality control material (QCM). Our mean results agree to 99% with the certified value of lead. The quality control material was supplied freeze-dried and reconstituted by adding 5 mL of water. The direct injection method was successfully applied for the determination of lead in blood samples that were collected from 78 different persons in two patient and control groups. The blood samples were diluted with deionized water in order to bring the concentration in the range of calibration curve. The concentration of lead was calculated using the calibration curve. To verify the validity of the method and in order to evaluate the recovery percentage (%), known amounts of lead including 5, 20 and 40 µg L^-1^ were spiked into the blood sample and good spike recoveries including 96.2%, 94.5%, and 97.3% were obtained, respectively. Furthermore, the accuracy of the method was confirmed by the analysis and determination of lead in QCM by using the calibration curve and considering the dilution factor. The concentration of lead found in QCM materials was very close to the certified value with the accuracy of 97.8%. The mean concentration of lead was found to be significantly lower (P = 0.0001) in control group (16.70 ± 12.51μg/dL) compared to addicts (57.04 ± 46.03μg/dL). When the addicts were divided into various age groups, there appeared to be a significant difference (P = 0.0451) in blood lead concentration as a function of age (Table 3), however when the control group was considered, no difference was observed (P = 0.51). Also, a tendency (P = 0.048) towards increasing BLL with respect to BMI was observed due to drug consumption, but there was no significant variation between BLL concentration and BMI when the control group was considered (P = 0.35) (Table 4).

## 4. Discussion

In this study, a comparison of oral/inhaled opium addicts with a healthy control group was investigated. Using the graphite furnace atomic absorption spectroscopy (GF-AAS) followed by chemometric analysis, sub-to-low µg L^-1^ concentrations of blood lead level (BLL) was detected in both the addict and the control groups. The patient group was selected through systematic incidental sampling from 150 orally or by inhalation opium-addicted patients referred to Shariati Hospital located in Tehran The drug-addict group (patient group) included 39 men with a mean age of 48.6 ± 7.3 years. The patient group was comprised of patients who used ingested/inhaled opium. The healthy control group was matched with drug-addict group in age and sex with the mean age of 44.8 ± 5.6 years. It was observed that the BLL in opium-addicts (57.04 ± 46.03μg/dL) was significantly higher than that of the healthy control group (16.70 ± 12.51μg/dL). The mean difference of both groups (t = 4.56) was statistically significant (P = 0.0001) suggesting that the opium addicts have an elevated BLL compared to the healthy group probably due to the addition of lead to the opium. The calibration graph was linear in a concentration range of 5-75 µg L^-1^ (R^2^ > 0.9980) with the detection limit of 1 µg L^-1^ level. An acceptable reproducibility (less than 2.50%) and good recoveries between 94-98 % were obtained. The accuracy of the method was also confirmed by the analysis of a quality control material (QCM).
